# The Role of Urodynamic Studies in Female Patients with Non-Neurogenic Lower Urinary Tract Dysfunction: A Retrospective Analysis

**DOI:** 10.5152/tud.2026.26035

**Published:** 2026-06-17

**Authors:** Hanan Mohammed Shamrani

**Affiliations:** Department of Obstetrics and Gynecology, King AbdulAziz University, Jeddah, Kingdom of Saudi Arabia

**Keywords:** Non-neurogenic lower urinary tract dysfunction, UDS, urodynamic studies, urology

## Abstract

**Objective:**

To evaluate the diagnostic role of urodynamic studies (UDS) in female lower urinary tract dysfunction (LUTD) and its correlation with clinical symptoms.

**Methods:**

A retrospective study design was followed, and 89 female patients were included (2020-2025). SPSS was used for descriptive and multivariate analyses of the association between obstetric history and pelvic surgeries with UDS findings.

**Results:**

Forty-three (48.3%), 18 (20.2%), and 8 (9%) had no comorbidities, hypertension (HTN), and diabetes mellitus, respectively. Sixteen (18%) had urological problems, 54 (60.7%) were grand multiparous, and 20 (22.5%) had cesarean section (C-section). The mean volumes at first sensation, first desire to void, and strong desire to void were 111.83 ± 48.43 mL, 184.36 ± 70.73 mL, and 289.24 ± 93.60 mL, respectively. Urodynamic study findings for the mean maximum cystometric capacity were 370.46 ± 102.20 mL, maximum flow rate of 41.42 ± 47.04 mL/sec, post-void residual volume of 137.25 ± 144.0 mL, voided volume of 378.86 ± 164.87 mL, Valsalva leak point pressure of 7.43 ± 44.73 cm/H_2_O, and detrusor pressure at capacity was 10.86 ± 18.97 cm/H_2_O. Most patients had normal bladder compliance; 2 (2.2%) reported reduced compliance. Urethral assessment indicated that only 4 (4.5%) had obstructive urethra, stress incontinence, and 4 (4.5%) correlated with UDS and clinical symptoms (*P* < .05). A non-significant association was found between storage, voiding symptoms with UDS outcomes, except for hesitancy (*P* = .01) and straining (*P* = .04). Multinomial logistic regression analysis indicated no significant correlation of obstetric history and pelvic surgeries with UDS.

**Conclusion:**

Urodynamic studies may be helpful in providing objective functional assessment in selected or complex cases. However, in the population, UDS did not consistently confirm these cases.

**Main Points**

- Bladder capacity and sensation parameters were within the expected ranges.
- Overall, most patients had normal UDS findings.
- Clinical symptoms demonstrated limited correlation with UDS findings.
- Prior pelvic surgeries and obstetric history were not significant predictors of UDS outcomes.

## Introduction

In urological practice, non-neurogenic lower urinary tract symptoms (LUTS) are most prevalent in females, as well as being an important presentation in females.^1^^,^^2^ According to the International Continence Society, this disease is categorized as urinary incontinence symptoms (stress urinary incontinence (SUI), mixed urinary incontinence (MUI), urgency urinary incontinence (UUI), postural incontinence, nocturnal enuresis), bladder storage symptoms (overactive bladder symptoms urgency, nocturia and frequency with or without urgency incontinence), voiding symptoms (urinary retention, straining to void, hesitancy, slow or interrupted stream, terminal dribble, splitting or spraying, position dependent micturition and incomplete emptying), post-micturition symptoms, and suspicious symptoms.^1^ In addition, UUI, mixed UI, and other symptoms, including hesitancy, slow stream, intermittency, splitting or spraying of the urinary stream, straining, and terminal dribble, are important indications.^3^ The prevalence of LUTS in females ranges from 11.8% to 88.5%, and this increasing risk can be attributed to certain factors, such as aging, work and marital status, alcohol consumption, comorbidities, vaginal delivery, higher parity, prolonged labor, instrumental delivery, post-menopausal status, and laceration.^4^ Similarly, lower urinary tract dysfunction (LUTD) is a closely related concept with almost similar symptoms to LUTS, and the International Continence Society defines it with storage (nighttime frequency, urge incontinence or urgency, daytime frequency), often encompassing symptoms of urethra, bladder, prostate in the case of males and the pelvic floor.^1^ In addition, voiding symptoms (prolonged micturition time or dysuria) are also included.^5^ Therefore, clinical examination of female patients with non-neurogenic LUTD is necessary for better management of symptoms.

Traditionally, female patients with LUTD mostly rely on a comprehensive medical history, quality of life questionnaires, physical examination, clinical questionnaires, voiding diary, and laboratory and para-clinical investigations.^6^ These tools play a critical role in the assessment of LUTD; however, these tools cannot often accurately characterize the main reasons, patients’ perceptions, or underlying mechanisms for the patient’s symptoms.^7^ Most importantly, symptom-based diagnosis may not be reliable in the diagnosis of LUTD in females, which can result in diagnostic uncertainty and potentially inappropriate treatment decisions.^8^ To overcome diagnostic uncertainty, other diagnostic assessment tools need to be explored, and among these tools, urodynamic studies (UDS) are considered the only method that can specify LUTD, providing more reliable and detailed information associated with LUTD, with minimum morbidity.^9^^,^^10^

Urodynamic studies is a standardized functional assessment tool used to measure the LUTD and successfully locate the underlying cause of LUTD, leading to a better understanding of the associated pathophysiology.^11^ Urodynamic studies is usually based on a series of clinical tests, like filling cystometry, uroflowmetry, pressure-flow studies, and urethral closure assessment, including measurement of the leak-point pressure and urethral pressure profilometry.^12^ It helps differentiate between impaired bladder compliance, detrusor overactivity, functional bladder outlet obstruction, SUI, and detrusor underactivity conditions that may present with similar clinical symptoms but require different management approaches.^13^^,^^14^ Clinical evidence has demonstrated that UDS retains significant relevance in females with LUTD, particularly in the case of urinary incontinence.^15^^-^^17^

Despite UDS being the gold standard for diagnosing LUTD, its utilization and findings in Saudi populations are poorly documented. Existing literature focuses predominantly on Western cohorts, limiting generalizability to Saudi women, who may exhibit unique risk profiles like higher parity and cultural barriers, such as reluctance to discuss pelvic symptoms, which delay diagnosis, leading to untreated morbidity. Therefore, this study aims to fill this gap by analyzing UDS findings in female patients at King Abdulaziz University Hospital, providing insights into local patterns of bladder dysfunction, assessing the accuracy of clinical symptom-based diagnosis compared to urodynamic confirmation, and correlating obstetric history (parity, vaginal delivery, pelvic organ prolapses) and pelvic surgeries with urodynamic findings.

### Primary Objective

To analyze UDS findings in female patients with LUTD at KAUH.

### Secondary Objective

To assess the accuracy of clinical symptom-based diagnosis compared to UDS confirmation. To correlate obstetric history (parity, vaginal delivery, pelvic organ prolapses) and pelvic surgeries with UDS findings.

### Hypothesis

It was hypothesized that specific clinical symptoms and obstetric history variables in patients with non-neurogenic LUTD would correlate significantly with abnormal UDS findings.

## Material and Methods

### Study Design

This study followed a retrospective study design for the investigation of female patients with LUTD who visited the King Abdulaziz University Hospital (KAUH) between 2020 and 2025. Medical records and urodynamic reports of included patients were reviewed retrospectively using hospital record databases.

### Selection Criteria

For the selection of study patients, certain inclusion criteria were set, such as females diagnosed with urinary incontinence or voiding dysfunction without any neurological disorders and who had completed UDS at King Abdulaziz University Hospital (KAUH) between 2020 and 2025. The LUTS were defined by the International Continence Society.

Exclusion criteria were also set for the selection of study patients, such as neurogenic bladder, male patients, and missing/incomplete UDS reports or symptom documentation.

### Patients Selection

Patients were identified through review of urodynamic laboratory records and outpatient databases and due to the limited number of patients, all 89 patient records were included in the present study.

### Urodynamic Studies Program

The UDS (MMT Water and Laborie Air) was used, which included the following study parameters: maximum flow rate assessment, voiding pressure flow study, filling cystometrography, urethral pressure profilometry, and sphincter electromyography (EMG) as per previously published techniques.^18^^,^^19^

### Data Collection

Data collection for this retrospective study involves patients who underwent UDS between 2020 and 2025. Data collection was performed by retrieving all relevant information from electronic medical records in the following domains: demographics, clinical background, UDS parameters, and outcomes. Since the study was not designed as a formal diagnostic accuracy investigation, rather it is defined as the ability of UDS to objectively characterize LUTD patterns in female patients and to support the association between clinical presentation and physiological bladder or urethral abnormalities.

### Ethical Consideration

Ethical approval was obtained from the Institutional Review Board, Unit of Biomedical Ethics, Research Ethics Committee, King Abdulaziz University, College of Medicine, Saudi Arabia with reference number 254-25. Ethical approval was obtained from the Institutional Review Board (IRB), with reference number 254-25. (Approval no: 254-25, Date: March 2, 2026). Patient information was retrieved from hospital electronic medical records, and due to the retrospective nature of the study, informed consent was waived. Only the principal investigator was allowed to access the patient’s medical records.

### Statistical Analysis

Descriptive statistics summarized patients’ demographic characteristics, clinical background, urodynamic findings, and outcomes in the form of frequency distributions, percentages, or mean with SD. Inferential statistics, including the chi-square test, were employed to examine relationships between demographic variables, clinical background, urodynamic findings, and outcomes. Multinomial logistic regression analysis was performed for the correlation of obstetric history and pelvic surgeries with urodynamic diagnosis findings. All analyses were performed using IBM SPSS for Windows (Version 22.0, IBM Corporation, Armonk, NY, USA) with a significance level set at *P* < .05. The graph was prepared using Microsoft Excel.

## Results

### Demographical Characteristics

After screening, 89 female patients, with a mean age of 53.91 ± 12.04 years, were included in the present retrospective analysis. The majority of patients were in the 41-50 and 51-60-year age groups (27, 30.3% and 27, 30.3%, respectively), with a statistically significant difference (*P* = .002). The mean body mass index (BMI) was 30.06 ± 4.91 kg/m^2^, indicating an overall obese population, with nearly half of the patients classified as obese (44, 49.4%), with significant distribution (*P* < .05). Most included patients had no comorbidities (43, 48.3%); however, 18 (20.2%) and 8 (9%) had hypertension (HTN) and diabetes mellitus, respectively (*P* < .05). Most patients had no history of urological problems (73, 82%), while only 16 (18%) had urological problems (*P* < .05). In terms of obstetric history, most included patients were grand multiparous (54, 60.7%), followed by multiparous (27, 30.3%), with only 4 (4.5%) nulliparous (*P* < .05). Previously, the vast majority of the patients had a vaginal delivery (86, 96.6%), while 20 (22.5%) reported cesarean section (C-section) (*P* < .05), as described in Table 1.

### Clinical Background

Upon physical examination, vaginal prolapse was identified in 38 (42.7%) patients, although this distribution was statistically non-significant (*P* = .16). Storage symptoms were highly prevalent, with urgency reported by 81 (91%) patients, incontinence by 80 (89.9%), daytime frequency exceeding 8 voids/day by 68 (75.3%), and nocturia >twice/night by 29 patients (32.6%), all demonstrating significant distribution (*P* < .05). Voiding symptoms were also commonly observed among included patients, including pain (32, 36%), slow stream (18, 20.2%), spraying (11, 12.4%), intermittency (24, 27%), hesitancy (20, 22.5%), straining (17, 19.1%), terminal dribbling (28, 31.5%), post-micturition dribbling (32, 36%), and a sensation of incomplete bladder emptying (57, 64%), each demonstrating statistically significant occurrence (*P* < .05). Furthermore, incontinence-specific clinical diagnosis indicated that MUI was the most common (40, 44.94%), followed by SUI (34, 38.2%), while MI (1, 1.1%) was the least reported incontinence (Table 2). Meanwhile, volume at first sensation was 111.83 ± 48.43mL, at first desire to void 184.36 ± 70.73 mL, and at strong desire to void 289.24 ± 93.60 mL, as indicated in Table 2.

Among prior treatments, Vesicare (24, 27%) and anterior pelvic repair (21, 23.6%) were the most frequently used treatments, while 8 (9%) patients had not received any treatment. Pre-urodynamic imaging/testing was performed in 10 (11.2%) patients, and 15 (16.9%) received antibiotic prophylaxis before undergoing urodynamic testing (*P* < .05), as described in Table 2.

### Urodynamic Study Parameters

The mean maximum cystometric capacity, maximum flow rate, post-void residual volume, voided volume, Valsalva leak point pressure, and detrusor pressure at capacity was 370.46 ± 102.20 mL, 41.42 ± 47.04 mL/sec, 137.25 ± 144.0 mL, 378.86 ± 164.87 mL, 7.43 ± 44.73 cm/H_2_O, and 10.86 ± 18.97 cm/H_2_O, respectively, indicating a substantial proportion of patients with incomplete bladder emptying and low storage-phase detrusor pressure (Table 3). Bladder compliance was found to be normal in most of the patients (87, 97.8%), while only 2 (2.2%, *P* < .05) reported reduced bladder compliance. Based on the urodynamic diagnosis, most patients had MUI (35, 39.3%), followed by UI (23, 25.8%), while 24 (27%) were found to be normal (Table 3).

**Figure 1:**
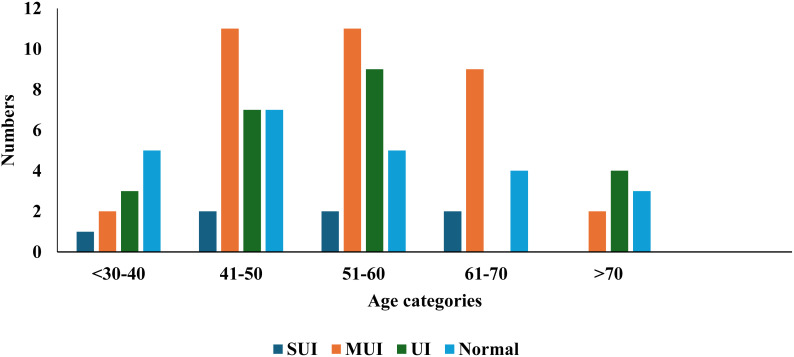
Age-wise distribution of urodynamic diagnosis (*P* = .38) UI, urinary incontinence; MUI, mixed urinary incontinence; SUI, stress urinary incontinence.

Moreover, age-wise distribution indicated that the 41-50 and 51-60 age groups had the highest number of patients with MUI and UI. However, the difference among groups was statistically non-significant (*P* = .38), as described in Figure 1.

### Outcomes

Most patients demonstrated a stable bladder (85, 95.5%) on urodynamic assessment, while 4 (4.5%) patients showed the features of an overactive bladder. Meanwhile, 89 (100%) patients demonstrated normal bladder contractility. Urethral assessment indicated that only 4 (4.5%) patients had obstructive urethra or stress incontinence, whereas 85 (95.5%) had no obstruction and were competent (Table 4). Furthermore, no adverse events were observed among patients, and only 4 (4.5%) patients had a correlation between urodynamic findings and clinical symptoms. Consistent with this, the predictive value of urodynamic was predominantly negative (85, 95.5%), with only 4 (4.5%) patients having a positive predictive value (*P* < .05), as described in Table 4.

### Accuracy of Clinical Symptom-Based Diagnosis Compared to Urodynamic Confirmation

Most storage and voiding symptoms, including vaginal prolapse, urgency, incontinence, increased daytime frequency, nocturia, pain, slow stream, spraying, intermittency, terminal dribbling, post-micturition dribbling, and sensation of incomplete emptying, did not show any significant (*P* > .05) association with urodynamic outcomes of SUI, MUI, UI or normal studies. In contrast, hesitancy (*P* = .01) and straining (*P* = .04) had a significant association with urodynamic diagnosis (Table 5).

### Multinomial Logistic Regression Analysis Between Obstetric History and Pelvic Surgeries with Urodynamic Diagnosis Findings

Overall, no significant association was observed between most obstetric variables and urodynamic diagnoses of SUI, MUI, or UUI, urodynamic diagnosis (bladder behavior), urodynamic diagnosis (urethra), urethral competence, or obstruction status. Individual predictors, like vaginal prolapse, parity classification, previous vaginal delivery, and previous C-section, had a non-significant association with any urodynamic outcomes (*P* > .05), as described in Table 6.

## Discussion

Urodynamic study findings in the present retrospective study revealed that most patients had a stable bladder, while only a few patients showed the features of an overactive bladder, obstructive urethra, and stress incontinence. A non-significant association was observed between age and UDS diagnosis. Meanwhile, a very limited number of patients had a correlation between UDS findings and clinical symptoms. Most clinical symptoms, including storage and voiding symptoms, did not show any significant association with UDS outcomes (SUI, MUI, UI). While hesitancy and straining had a significant association with UDS diagnosis. Multinomial logistic regression analysis also indicated a non-significant correlation between obstetric history and pelvic surgeries with UDS diagnosis findings. The findings should be interpreted with caution, given the multiple comparisons performed and the lack of significance in multinomial logistic regression analysis. Due to the low explanatory power of the regression model, analyzed variables did not contribute to the variability in UDS outcomes.

Similar findings were observed in another retrospective study performed on Chinese females, and most of the females were found with a normo-active detrusor/sphincter (927, 28.4%), while 20.8% were found with idiopathic detrusor overactivity and 19.9% cases of detrusor overactivity. In addition, storage (62.5%) and voiding (16.5%) symptoms were also observed in the included patients.^20^ In contrast, another retrospective study included 401 patients who underwent UDS; most of the patients were diagnosed with detrusor underactivity (23.2%) and bladder outlet obstruction (14.5%). Meanwhile, a significant difference was observed between age and UDS diagnosis (*X*^2^ = 1899.91, *P* < .001).^21^ Haylen, Krishnan^22^ included 592 females in a urogynecological assessment via UDS, and 39% females had voiding difficulty, 72% had USI, 61% had uterine/vaginal prolapse, and 13% had overactive bladder. It needs to be understood that peak flow of <15 mL/s and detrusor pressure in excess of 60 cm of water should be considered and accepted as obstruction in females, provided that any video evidence of relaxation of the urethral sphincter and funneling of the bladder neck during voiding.^23^ A systematic review and meta-analysis with 8 randomized trials demonstrated non-significant efficacy of UDS in the diagnosis of LUTD (relative risk (RR), 1.00, 95% CI; 0.93-1.07).^10^ Another retrospective study analyzed 1737 reports and outcomes demonstrated the inverse, but with a weak association between SUI and bladder outlet, storage symptoms, and cystometric capacity, and a moderate association with urine leak.^24^ Meanwhile, a multicenter, pragmatic, and 2-arm UPSTREAM study demonstrated that UDS before surgery for LUTS resulted in a better diagnosis and was found to be superior as a routine care pathway.^25^ Questions can arise as to why UDS is necessary to be performed in females with LUTD, and a possible explanation can be that UDS objectively evaluates the functions associated with the bladder and urothelial, and, most importantly, UDS helps to provide details and accurately identify the pathophysiology responsible for symptoms of LUTD.^26^ Another reason can be that female LUTD is often multifactorial, and clinical symptoms overlap, and clinical presentation alone cannot reliably distinguish between bladder outlet obstruction, detrusor overactivity, detrusor underactivity, or SUI.^27^ Before 1980, UDS was used to consider that accurately diagnosed SUI patients were the only or ideal candidates for successful surgery.^28^ Now, according to the European expert consensus, diagnosis through UDS is helpful for all patients with initial treatment failure and should not be skipped.^29^ This is well-supported by the findings of a prospective study, which included 687 female patients who received treatment and, according to the UDS findings, demonstrated a significant (*P* = .02) improvement in the symptoms associated with bladder compared to the non-UDS group (57% vs 45%).^30^

Clinically, UDS provides useful insights for clinicians to differentiate between SUI, detrusor overactivity, or voiding dysfunction, and thereby can facilitate individualized and evidence-based planning for treatment. Even normal UDS findings, which were observed in the findings, can carry clinical relevance by reassuring both clinician and patient, and by supporting behavioral and conservative management approaches. Collectively, selective use of UDS in the case of a patient with a complex presentation can optimize the decision-making process and promote a more rational use of available management strategies, ultimately resulting in improved clinical outcomes.

Prior to interpreting the outcomes, certain limitations should be considered, such as the retrospective nature of this study and the fact that it was performed in a single center, which limits the generalizability of the outcomes. Another limitation is the small sample size, and the symptom assessment was based on clinical documentation, without inclusion of standardized symptom severity scores or valid questionnaires, which may affect the accuracy of the correlation between clinical presentation and UDS findings. Future prospective, multicenter, and longitudinal studies are required to validate the outcomes of the present study.

## Conclusion

The findings demonstrated that most patients had a stable bladder, and a few showed overactive bladder on UDS assessment, and urethral assessment indicated that a few patients had obstructive urethra and stress incontinence. A minimal number of patients had a correlation between UDS findings and clinical symptoms and showed a positive predictive value. Most features of clinical symptoms had not been significantly associated with UDS findings, except for hesitancy and straining, which had a significant association with UDS diagnosis. Multinomial logistic regression analysis also indicated a non-significant correlation of obstetric history and pelvic surgeries with UDS findings of LUTD in females. Future longitudinal studies are required to validate the findings of the present study.

## Figures and Tables

**Table 1.** Demographical Characteristics of Included Study Participants

Variables	Frequency	Percentage	*P*
Age (years) (mean ± SD)	53.91 ± 12.04		
Age categories (years)			
<30-40	11	12.4	.002
41-50	27	30.3	
51-60	27	30.3	
61-70	15	16.9	
>70	9	10.1	
BMI (kg/m^2^) (mean ± SD)	30.06 ± 4.91		
BMI (kg/m^2^) categories			
Underweight (<18.5)	1	1.1	<.05
Normal weight (18.5-24.9)	12	13.5	
Overweight (25.0-29.9)	32	36	
Obesity (≥30.0)	44	49.4	
Comorbidities			
HTN	18	20.2	<.05
DM	8	9	
Multiple (HTN/DM/DLP)	10	11.2	
Others (ITP, hypothyroidism, bronchial asthma, IDA, HLD, BA, GERD)	10	11.2	
No	43	48.3	
Urology history			
Yes	16	18	<.05
No	73	82	
Parity classification			
Nulliparous (P0)	4	4.5	<.05
Primiparous (P1)	4	45	
Multiparous (P2-4)	27	30.3	
Grand multiparous (P ≥ 5)	54	60.7	
Previous vaginal delivery			
Yes	86	96.6	<.05
No	3	3.4	
Previous cesarean section			
Yes	20	22.5	<.05
No	69	77.5	

DM, diabetes mellitus; DLP, detrusor leak point pressure; GERD, gastroesophageal reflux disease; HTN, hypertension; IDA, iron deficiency anemia; SD, standard deviation.

**Table 2.** Clinical Background of Included Patients

Variables	Frequency	Percentage	*P*
Physical examination			
Vaginal prolapse			
Yes	38	42.7	.16
No	51	57.3	
Storage symptoms			
Urgency			
Yes	81	91	<.05
No	8	9	
Incontinence			
Yes	80	89.9	<.05
No	9	10.1	
Frequency >8×/day			
Yes	68	75.3	<.05
No	22	24.7	
Nocturis >2×/night			
Yes	29	32.6	<.05
No	60	67.4	
Voiding symptoms			
Pain			
Yes	32	36	<.05
No	57	64	
Slow stream			
Yes	18	20.2	<.05
No	71	79.8	
Spraying			
Yes	11	12.4	<.05
No	78	87.6	
Intermittency			
Yes	24	27	<.05
No	65	73	
Hesitancy			
Yes	20	22.5	<.05
No	69	77.5	
Straining			
Yes	17	19.1	<.05
No	72	80.9	
Terminal dribbling			
Yes	28	31.5	<.05
No	61	68.5	
Post-micturition dribbling			
Yes	32	36	<.05
No	57	64	
Incomplete emptying			
Yes	57	64	<.05
No	32	36	
Incontinence-specific clinical diagnosis			
Indication for UDS			
MUI	39	44.94	<.05
SUI	34	38.2	
UI	12	13.5	
SI	3	3.4	
Previous treatment			
Vesicare	24	27	<.05
Anterior pelvic repair	21	23.6	
Kegels exercise	9	10.1	
Pelvic floor exercise	9	10.1	
No treatment	8	9	
Others	18	20.2	
Pre-UDS imaging/testing			
Yes	10	11.2	<.05
No	79	88.8	
Pre-UDS antibiotic prophylaxis			
Yes	15	16.9	<.05
No	74	83.1	
Volume at first sensation (mean ± SD)	111.83 ± 48.43		
Volume at first desire to void (mean ± SD)	184.36 ± 70.73		
Volume at strong desire to void (mean ± SD)	289.24 ± 93.60		

MUI, mixed urinary incontinence; SUI, stress urinary incontinence; UI, urinary incontinence; UDS, urodynamic studies.

**Table 3.** Characteristics of the Urodynamic Study Parameters of Included Patients

Variables	Frequency	Percentage	*P*
Maximum cystometric capacity (Vmax) (mL)	370.46 ± 102.20		
Maximum flow rate (Qmax) (mL/sec)	41.42 ± 47.04		
Post-void residual volume (mL)	137.25 ± 144		
Voided volume	378.86 ± 164.87		
Valsalva leak point pressure (cm/H_2_O)	7.43 ± 44.73		
Detrusor pressure at capacity (cm H_2_O)	10.86 ± 18.97		
Bladder compliance			
Normal	87	97.8	<.05
Reduced	2	2.2	
Urodynamic diagnosis			
MUI	35	39.3	<.05
UI	23	25.8	
SUI	7	7.9	
Normal	24	27	

MUI, mixed urinary incontinence; SUI, stress urinary incontinence; UI, urinary incontinence; UDS, urodynamic studies.

**Table 4.** Post-Study Outcomes of Included Patients

Variables	Frequency	Percentage	*P*
Urodynamic diagnosis (bladder)			
Stable	85	95.5	<.05
Overactive	4	4.5	
Urodynamic diagnosis bladder (normal/contractility underactive)			
Normal	89	100	Not performed
Urodynamic diagnosis (urethra)			
Competent	85	95.5	<.05
Stress incontinence	4	4.5	
Urodynamic diagnosis (urethra)			
Unobstructed	85	95.5	<.05
Obstructed	4	4	
Adverse events			
No	89	100	Not performed
Correlation of UDS findings with symptoms			
Yes	4	4.5	<.05
No	85	95.5	
Predictive value			
Negative	85	95.5	<.05
Positive	4	4.5	

UDS, urodynamic studies.

**Table 5.** Comparison of Clinical Symptom-Based Diagnosis and Urodynamic Diagnosis

Clinical Symptoms-Based Diagnosis	Urodynamic Diagnosis				*P*
	SUI	MUI	UI	Normal	
Vaginal prolapse					
Yes	4	18	7	9	.33
No	3	17	16	15	
Urgency					
Yes	6	33	21	21	.78
No	1	2	2	3	
Incontinence					
Yes	7	31	20	22	.76
No	0	4	3	2	
Frequency >8x/day					
Yes	7	24	17	19	.33
No	0	11	6	5	
Nocturia >2x/night					
Yes	1	11	11	6	.24
No	6	24	12	18	
Pain					
Yes	2	13	10	7	.74
No	5	22	13	17	
Slow stream					
Yes	1	9	6	2	.33
No	6	26	17	22	
Spraying					
Yes	0	7	3	1	.22
No	7	28	20	23	
Intermittency					
Yes	1	14	5	4	.15
No	6	21	18	20	
Hesitancy					
Yes	0	12	7	1	.01
No	7	23	16	23	
Straining					
Yes	0	10	6	1	.04
No	7	25	17	23	
Terminal dribbling					
Yes	0	12	11	5	.05
No	7	23	12	19	
Post-micturition dribbling					
Yes	1	15	10	6	.26
No	6	20	13	18	
Incomplete emptying					
Yes	3	23	16	15	.62
No	4	12	7	9	

MUI, mixed urinary incontinence; SUI, stress urinary incontinence; UI, urinary incontinence.

**Table 6.** A Multinomial Logistic Regression Analysis Of Obstetric History And Pelvic Surgeries With Urodynamic Diagnosis Findings

Source	Dependent Variable	B	SD	Wald	*df*	Sig	Exp (B)	LB	UB
Vaginal prolapse	Intercept	0.05	0.38	0.02	1	0.88			
	Urodynamic diagnosis (SUI, MUI, UUI)	−0.21	0.16	1.62	1	0.20	0.80	0.58	1.12
	Urodynamic diagnosis (bladder)	1.53	1.18	1.67	1	0.19	4.64	0.45	47.31
	Urodynamic diagnosis (bladder (normal contractility, underactive))	0			0				
	Urodynamic diagnosis (urethra (competent stress, incontinence))	0			0				
	Urodynamic diagnosis (urethra (unobstructed, obstructed))	0			0				
Parity classification									
Nulliparous	Intercept	−2.33	0.88	6.94	1	0.008			
	Urodynamic diagnosis (SUI, MUI, UUI)	−0.11	0.40	0.08	1	0.77	0.89	0.40	1.97
	Urodynamic diagnosis (bladder)	−17.2	0.00		1		3.07	3.07	3.07
	Urodynamic diagnosis (bladder (normal contractility, underactive))	0			0				
	Urodynamic diagnosis (urethra (competent stress, incontinence))	0			0				
	Urodynamic diagnosis (urethra (unobstructed, obstructed))	0			0				
Primiparous	Intercept	−2.60	0.92	7.89	1	0.005			
	Urodynamic diagnosis (SUI, MUI, UUI)	0.02	0.38	0.006	1	0.93	1.03	0.48	2.18
	Urodynamic diagnosis (bladder)	−17.3	0.00		1		2.91	2.91	2.91
	Urodynamic diagnosis (bladder (normal contractility, underactive))	0			0				
	Urodynamic diagnosis (urethra (competent stress, incontinence))	0			0				
	Urodynamic diagnosis (urethra (unobstructed, obstructed))	0			0				
Multiparous	Intercept	−0.80	0.42	3.50	1	0.06			
	Urodynamic diagnosis (SUI, MUI, UUI)	0.06	0.17	0.13	1	0.71	1.06	0.75	1.50
	Urodynamic diagnosis (bladder)	−0.44	1.18	0.14	1	0.70	0.64	0.06	6.50
	Urodynamic diagnosis (bladder (normal contractility, underactive))	0			0				
	Urodynamic diagnosis (urethra (competent stress, incontinence))	0			0				
	Urodynamic diagnosis (urethra (unobstructed, obstructed))	0			0				
Previous vaginal delivery	Intercept	3.77	1.13	10.69	1	0.001			
	Urodynamic diagnosis (SUI, MUI, UUI)	−0.19	0.42	0.21	1	0.64	0.82	0.36	1.87
	Urodynamic diagnosis (bladder)	17.21	0.00		1		2.72	2.72	2.72
	Urodynamic diagnosis (bladder (normal contractility, underactive))	0			0				
	Urodynamic diagnosis (urethra (competent stress, incontinence))	0			0				
	Urodynamic diagnosis (urethra (unobstructed, obstructed))	0			0				
Previous C-section	Intercept	−0.71	0.44	2.62	1	0.10			
	Urodynamic diagnosis (SUI, MUI, UUI)	−0.25	0.20	1.50	1	0.22	0.77	0.52	1.16
	Urodynamic diagnosis (bladder)	−19.3	0.00		1		3.80	3.80	3.80
	Urodynamic diagnosis (bladder (normal contractility, underactive))	0			0				
	Urodynamic diagnosis (urethra (competent stress, incontinence))	0			0				
	Urodynamic diagnosis (urethra (unobstructed, obstructed))	0			0				

MUI, mixed urinary incontinence; SUI, stress urinary incontinence; UUI, urgency urinary incontinence.

## Data Availability

The data that support the findings of this study are available on request from the corresponding author.

## References

[1] ICS. Assessment of lower urinary tract symptoms. International Continence Society, UK. Accessed 14 May, 2026. https://www.ics.org/public/factsheets/assessmentoflowerurinarytractsymptoms.

[2] Arlandis S Bø K Cobussen-Boekhorst H European Association of Urology guidelines on the management of female non-neurogenic lower urinary tract symptoms. Part 2: underactive bladder, bladder outlet obstruction, and nocturia. Eur Urol. 2022;82(1):60 70. (doi: 10.1016/j.eururo.2022.01.044) 35181193

[3] Harding CK Lapitan M Arlandis S Non-neurogenic female. Low Urin Tract Symptoms (LUTS). European Association of Urology. 2024 Update.

[4] Tahra A Bayrak Ö Dmochowski R. The epidemiology and population-based studies of women with lower urinary tract symptoms: a systematic review. Turk J Urol. 2022;48(2):155 165. (doi: 10.5152/tud.2022.21325) 35420059 PMC9612779

[5] Xu D Han S Wang J Relationship between lower urinary tract dysfunction and clinical features in Chinese Parkinson's disease patients. Parkinsons Dis. 2019;2019:1 7. (doi: 10.1155/2019/6820937) PMC6425341 30949327

[6] Ivanov M Ceban E. The importance of multimodal diagnostic methods for therapeutic decision making for overactive bladder in women. Med Pharm Rep. 2025;98(2):165 175. (doi: 10.15386/mpr-2669) 40371413 PMC12070906

[7] Skorupska K Kamińska A. Advantages and disadvantages of commonly used urinary incontinence questionnaires—how to correctly choose questionnaire in urinary incontinence diagnosis? J Clin Med. 2025;14(22):8196. (doi: 10.3390/jcm14228196) PMC12653568 41303232

[8] Kuo HC. Clinical symptoms are not reliable in the diagnosis of lower urinary tract dysfunction in women. J Formos Med Assoc. 2012;111(7):386 391. (doi: 10.1016/j.jfma.2011.05.014) 22817816

[9] Al Mousa RT Al Dossary N Hashim H. The role of urodynamics in females with lower urinary tract symptoms. Arab J Urol. 2019;17(1):2 9. (doi: 10.1080/2090598X.2019.1589931) 31258939 PMC6583751

[10] Bodmer NS Wirth C Birkhäuser V Randomised controlled trials assessing the clinical value of urodynamic studies: a systematic review and meta-analysis. Euro Urol Open Sci. 2022;44:131 141. (doi: 10.1016/j.euros.2022.08.013) PMC9469658 36110903

[11] Schäfer W Abrams P Liao L Good urodynamic practices: uroflowmetry, filling cystometry, and pressure-flow studies. Neurourol Urodyn. 2002;21(3):261 274. (doi: 10.1002/nau.10066) 11948720

[12] Yamanishi T Sakakibara R Uchiyama T Role of urodynamic studies in the diagnosis and treatment of lower urinary tract symptoms. Urol Sci. 2011;22(3):120 128. (doi: 10.1016/j.urols.2011.08.007)

[13] Groutz A Blaivas JG. Non-neurogenic female voiding dysfunction. Curr Opin Urol. 2002;12(4):311 316. (doi: 10.1097/00042307-200207000-00009) 12072652

[14] Chen S-F Lee C-L Kuo H-C. Change of detrusor Contractility in Patients with and without Bladder Outlet Obstruction at Ten or More Years of follow-up. Sci Rep. 2019;9(1):18887. (doi: 10.1038/s41598-019-55386-2) PMC6906492 31827203

[15] Rosier PF. The evidence for urodynamic investigation of patients with symptoms of urinary incontinence. F1000prime reports. 2013;5:8. 10.12703/P5-8 PMC3590786 23513180

[16] Clement KD Lapitan MC Omar MI Urodynamic studies for management of urinary incontinence in children and adults. Cochrane Database Syst Rev. 2013;2013(10):Cd003195. (doi: 10.1002/14651858.CD003195.pub3) PMC6599826 24166676

[17] Baines G Da Silva AS Araklitis G Recent advances in urodynamics in women. F1000Research; vol 9; 2020. 10.12688/f1000research.24640.1 PMC7308832 32595939

[18] Xu D Qu C Meng H Dysfunctional voiding confirmed by transdermal perineal electromyography, and its effective treatment with baclofen in women with lower urinary tract symptoms: a randomized double-blind placebo-controlled crossover trial. BJU Int. 2007;100(3):588 592. (doi: 10.1111/j.1464-410X.2007.06987.x) 17511770

[19] Xu DF Zhang S Wang CZ Low-frequency electrotherapy for female patients with detrusor underactivity due to neuromuscular deficiency. Int Urogynecol J. 2012;23(8):1007 1015. (doi: 10.1007/s00192-012-1714-2) 22441580 PMC3396337

[20] Wang L Wang C Qu C Relationship between urodynamic patterns and lower urinary tract symptoms in Chinese women with a non-neurogenic bladder. Asian J Urol. 2016;3(1):10 19. (doi: 10.1016/j.ajur.2015.11.004) 29264157 PMC5730814

[21] Khadga A Adhikari MB Maharjan B Elucidating the pivotal role of urodynamic studies in diagnosing and managing lower urinary tract symptoms: insights from a retrospective observational study at a single Nepalese center. Int J Surg Open. 2025;63(6):451 457. (doi: 10.1097/IO9.0000000000000323)

[22] Haylen BT Krishnan S Schulz S Has the true prevalence of voiding difficulty in urogynecology patients been underestimated? Int Urogynecol J. 2007;18(1):53 56. (doi: 10.1007/s00192-006-0094-x) 16596458

[23] Diokno AC Hollander JB Bennett CJ. Bladder neck obstruction in women: a real entity. J Urol. 1984;132(2):294 298. (doi: 10.1016/S0022-5347(17)49601-7) 6539828

[24] Al-Zahrani AA Gajewski J. Urodynamic findings in women with refractory overactive bladder symptoms. Int J Urol: Off J Jpn Urol Assoc. 2016;23(1):75 79. (doi: 10.1111/iju.12954) 26417863

[25] Drake MJ Lewis AL Young GJ Diagnostic assessment of lower urinary tract symptoms in men considering prostate surgery: a noninferiority randomised controlled trial of urodynamics in 26 hospitals. Eur Urol. 2020;78(5):701 710. (doi: 10.1016/j.eururo.2020.06.004) 32616406

[26] Kim SJ Choo HJ Yoon H. Diagnostic value of the maximum urethral closing pressure in women with overactive bladder symptoms and functional bladder outlet obstruction. Int Neurourol J. 2022;26(suppl 1):S1 S7. (doi: 10.5213/inj.2040482.241) 35236047 PMC8896775

[27] Garbas K Zapała Ł Ślusarczyk A Cracking the LUTS Code: a Pre-Urodynamic Tool for DU vs. BOO Diagnosis in Female Patients with Non-Neurogenic LUTS. J Clin Med. 2025;14(11):3674. (doi: 10.3390/jcm14113674) PMC12156388 40507437

[28] Cardozo LD Stanton SL. Genuine stress incontinence and detrusor instability--a review of 200 patients. BJOG. 1980;87(3):184 190. (doi: 10.1111/j.1471-0528.1980.tb04515.x) 7387918

[29] Finazzi-Agro E Gammie A Kessler TM Urodynamics useless in female stress urinary incontinence? Time for some sense—a European expert consensus. Eur Urol Focus. 2020;6(1):137 145. (doi: 10.1016/j.euf.2018.07.031) 30061075

[30] Verghese TS Middleton LJ Daniels JP The impact of urodynamics on treatment and outcomes in women with an overactive bladder: a longitudinal prospective follow-up study. Int Urogynecol J. 2017;29(4):513 519. (doi: 10.1007/s00192-017-3414-4) 28721482 PMC5876271

